# First person – Sol Díaz de León-Guerrero

**DOI:** 10.1242/dmm.049442

**Published:** 2022-06-13

## Abstract

First Person is a series of interviews with the first authors of a selection of papers published in Disease Models & Mechanisms, helping early-career researchers promote themselves alongside their papers. Sol Díaz de León-Guerrero is first author on ‘ An enriched environment re-establishes metabolic homeostasis by reducing obesity-induced inflammation’, published in DMM. Sol is a PhD student in the lab of Dr Leonor Pérez-Martínez at Universidad Nacional Autónoma de México, Morelos, Mexico, investigating how brain function is affected during pathological conditions.


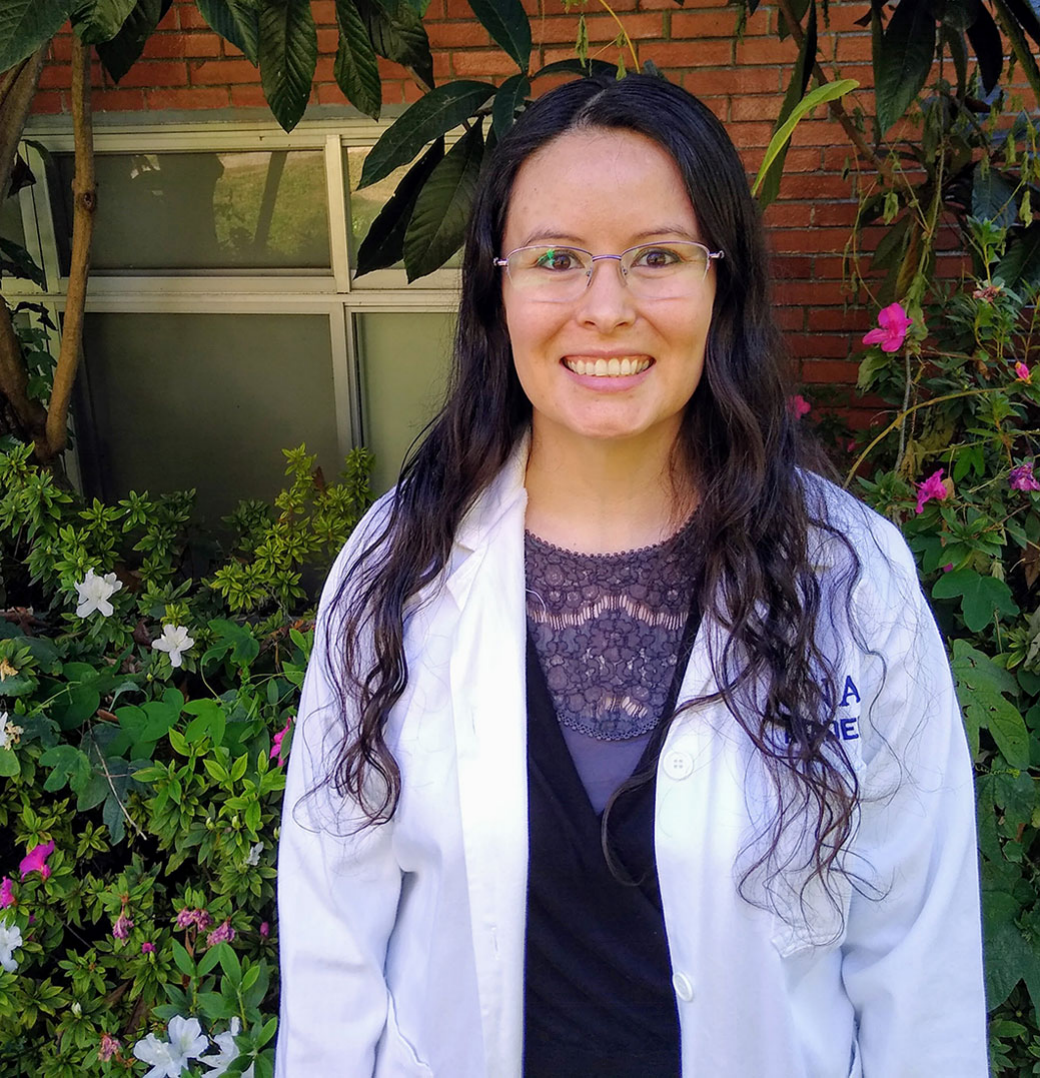


**Sol Díaz de León-Guerrero**

**How would you explain the main findings of your paper to non-scientific family and friends?**

Obesity has been increasing steadily in many countries over the last decades. This poses an important health issue as it increases the risk of developing other conditions such as cardiovascular diseases and type II diabetes. Obesity causes inflammation in different tissues, such as the pancreas, liver, adipose tissue and even the brain, impairing their normal function. We wanted to study a non-invasive therapeutic approach to treat obesity and insulin resistance by studying the effects of environmental enrichment. Normally, laboratory mice are housed in plastic cages with free access to food and water but no other source of stimulus. An enriched environment consists of housing conditions that promote cognitive, social and physical stimulation. This is done by keeping mice in larger cages filled with different kinds of toys, and by allowing a larger number of animals to be housed together. The enriched environment has been studied for many years and has been found to have beneficial effects in the brain, such as improving learning and memory, as well as promoting the generation of new neurons. Additionally, recent studies have also shown that it can regulate the immune system. For our research, mice were given a diet with high-fat and -caloric content to induce obesity and insulin resistance. Once they had metabolic alterations, we switched them to live in an enriched environment, keeping the same diet. We found that the change in housing conditions improved the metabolism of mice even if they were still overweight. We also saw that the enriched environment reduced damage to the liver and the adipose tissue caused by obesity, and lowered inflammation in the adipose tissue and in the brain.

“[…] an enriched environment could be used as a therapeutic approach to treat the metabolic alterations caused by obesity.”

**What are the potential implications of these results for your field of research?**

Our results suggest that an enriched environment could be used as a therapeutic approach to treat the metabolic alterations caused by obesity. We found that an enriched environment was capable of decreasing inflammation in the brain as well as in the periphery in our model of diet-induced obesity. Given that an inflammatory process has been implicated in the development of many diseases and in ageing, it would be of interest to study the effects of an enriched environment in other pathologies, and especially in those that have an inflammatory component.

**What are the main advantages and drawbacks of the model system you have used as it relates to the disease you are investigating?**

Murine models have been used extensively to study the development of obesity and type II diabetes by using both diet-induced and genetic models. Feeding mice with a high-fat diet leads to obesity, generating an inflammatory process that impairs normal tissue function and promotes the development of insulin resistance. This resembles what happens in humans, where overnutrition and the intake of food with a high caloric content is one of the main causes of obesity.

Regarding the enriched environment, its effects can be easily studied in animal models given that it differs greatly from the standard housing conditions that are normally used. However, in the case of humans, it is harder to determine what could be considered as environmental enrichment as we already live very complex lifestyles. Further research needs to be done to determine how best to translate the aspects of an enriched environment into clinical settings to help treat different pathologies.

**Figure:**
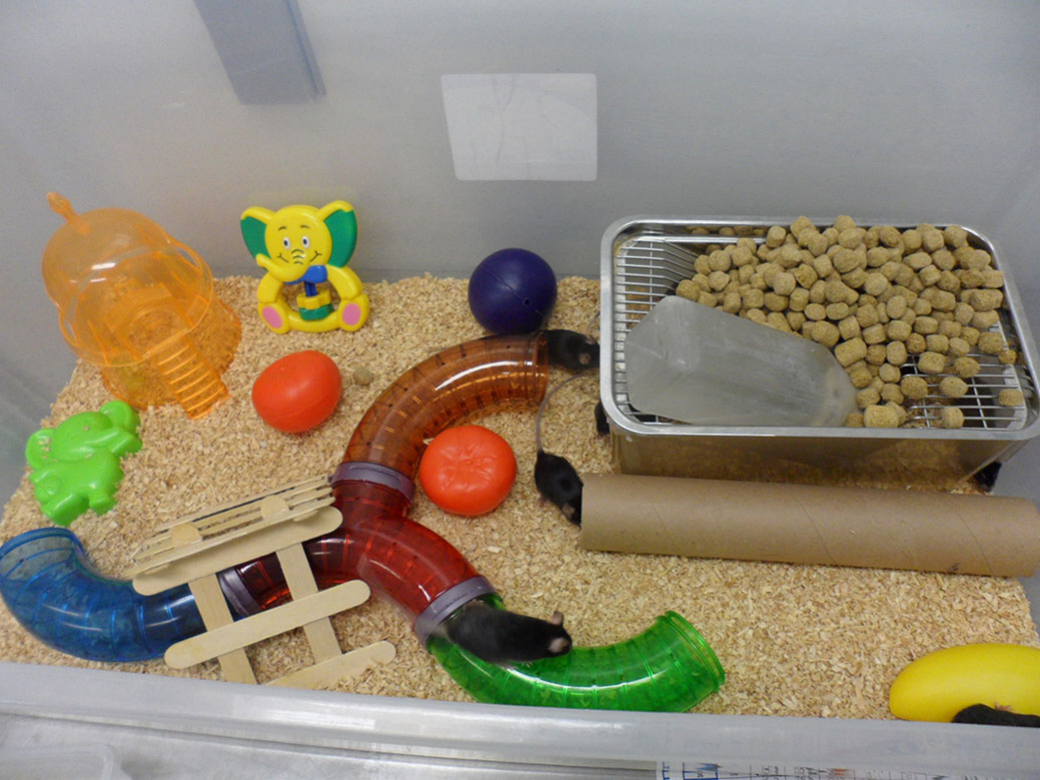
Enriched environment housing conditions containing different toys and a higher number of mice per cage.

**What has surprised you the most while conducting your research?**

When we started designing this project we expected that the enriched environment would promote weight loss in our obese mice. However, we found that the enriched environment improved glucose tolerance and decreased inflammation even in mice that were still overweight. This might help us get some insight into how some people can still be overweight but metabolically healthy.

**Describe what you think is the most significant challenge impacting your research at this time and how will this be addressed over the next 10 years?**

Current estimates show that the percentage of people with obesity will keep increasing in most countries. This shows the importance of research in this area to understand the genetic and molecular mechanisms underlying this pathology to further identify therapeutic targets. I believe that in the next years we will be able to identify genetic and environmental risk factors for each individual to personalize treatment.

“[…] increasing job opportunities and funding options would greatly benefit early-career scientists, especially to help them remain in academia.”

**What changes do you think could improve the professional lives of early-career scientists?**

I think that increasing job opportunities and funding options would greatly benefit early-career scientists, especially to help them remain in academia. I believe that it is very important to give new scientists the opportunity to develop their own research lines and lead their own labs, which can bring new insights and perspectives into existing fields.

**What's next for you?**

For now, my biggest priority is graduating from my PhD early next year. Afterwards, I hope to continue my career in science and learn more about how disease impacts brain function.
